# Genome-wide mRNA and miRNA expression profiling reveal multiple regulatory networks in colorectal cancer

**DOI:** 10.1038/cddis.2014.556

**Published:** 2015-01-22

**Authors:** R Vishnubalaji, R Hamam, M-H Abdulla, M A V Mohammed, M Kassem, O Al-Obeed, A Aldahmash, N M Alajez

**Affiliations:** 1 Stem Cell Unit, Department of Anatomy, College of Medicine, King Saud University, Riyadh 11461, Kingdom of Saudi Arabia; 2 Colorectal Research Center, Department of Surgery, King Khalid University Hospital, College of Medicine, King Saud University, Riyadh, Kingdom of Saudi Arabia; 3 KMEB, Department of Endocrinology, University of Southern Denmark, Odense, Denmark; 4 Danish Stem Cell Center (DanStem), Panum Institute, University of Copenhagen, Copenhagen, Denmark

## Abstract

Despite recent advances in cancer management, colorectal cancer (CRC) remains the third most common cancer and a major health-care problem worldwide. MicroRNAs have recently emerged as key regulators of cancer development and progression by targeting multiple cancer-related genes; however, such regulatory networks are not well characterized in CRC. Thus, the aim of this study was to perform global messenger RNA (mRNA) and microRNA expression profiling in the same CRC samples and adjacent normal tissues and to identify potential miRNA-mRNA regulatory networks. Our data revealed 1273 significantly upregulated and 1902 downregulated genes in CRC. Pathway analysis revealed significant enrichment in cell cycle, integrated cancer, Wnt (wingless-type MMTV integration site family member), matrix metalloproteinase, and TGF-*β* pathways in CRC. Pharmacological inhibition of Wnt (using XAV939 or IWP-2) or TGF-*β* (using SB-431542) pathways led to dose- and time-dependent inhibition of CRC cell growth. Similarly, our data revealed up- (42) and downregulated (61) microRNAs in the same matched samples. Using target prediction and bioinformatics, ~77% of the upregulated genes were predicted to be targeted by microRNAs found to be downregulated in CRC. We subsequently focused on EZH2 (*enhancer of zeste* homolog 2 ), which was found to be regulated by hsa-miR-26a-5p and several members of the let-7 (lethal-7) family in CRC. Significant inverse correlation between EZH2 and hsa-miR-26a-5p (*R*^2^=0.56, *P*=0.0001) and hsa-let-7b-5p (*R*^2^=0.19, *P*=0.02) expression was observed in the same samples, corroborating the belief of EZH2 being a *bona fide* target for these two miRNAs in CRC. Pharmacological inhibition of EZH2 led to significant reduction in trimethylated histone H3 on lysine 27 (H3K27) methylation, marked reduction in cell proliferation, and migration *in vitro*. Concordantly, small interfering RNA-mediated knockdown of EZH2 led to similar effects on CRC cell growth *in vitro*. Therefore, our data have revealed several hundred potential miRNA-mRNA regulatory networks in CRC and suggest targeting relevant networks as potential therapeutic strategy for CRC.

Colorectal cancer (CRC) is among the most prevalent types of cancers causing high mortality rates around the world. CRC is the third most common type of cancer (1.23 million, 9.7% up to 2010) in both genders (males (10%) and females (9.4%)), with the highest rates in New Zealand, Australia, and Western Europe.^1^ CRC has been reported as the fourth most common malignancy (male 7.6%, female 8.6%), particularly in Central and Eastern Europe where it has been reported for the highest mortality rate in both genders.^1^ In eastern Asia, countries such as Japan, China, South Korea, and Singapore experienced a two- to four fold increase in CRC incidence in the past few decades.^2^ Similar trends have been observed in Saudi Arabia.^3^ In the Kingdom of Saudi Arabia, CRC is the most common cancer form among men and the second most common cancer among women, where 66.1% of CRC incident cases were reported in males and 33.9% in women.^4^ The 5-year overall survival was reported to be 63.3% for patients with localized disease, 50.2% for those with regional disease, and 14.7% for patients with distant metastases.^4^ In developing countries, the 5-year survival rates for CRC range from 28 to 42%.^5^

The conventional chemotherapy for CRC involves the use of highly toxic drugs with many undesirable side effects,^6, 7^ underscoring the need to identify novel biomarkers for early diagnosis and for better disease stratification and treatment choices. Recently, microRNAs (miRNAs) have emerged as having a key role in cancer development, progression, and resistance to chemotherapy.^8, 9, 10^ miRNAs are noncoding small RNAs (20–22 nucleotides), which possess posttranscriptional regulatory functions in diverse biological processes such as proliferation, apoptosis, cell cycle regulation, stem cell maintenance, differentiation, development, metabolism, and aging.^8, 11, 12, 13, 14,15^ A variety of methods have been used ranging from miRNA microarrays to global miRNA expression profiling with deep sequencing to determine the expression pattern of miRNAs in cancer tissues.^16, 17^ Abnormal expression of miRNAs has been associated with a large number of human cancers. In CRC, a number of studies have reported altered miRNA expression pattern, suggesting a plausible role for the aberrant miRNA expression in CRC biology.^17, 18^ The natural mechanisms for the dysregulation of miRNAs are still largely unknown, although gene amplification, genomic loss, and promoter hypermethylation have been reported as the potential mechanisms in various cancers.^14, 19, 20^ Strategies based on restoration of downregulated miRNAs or inhibition of upregulated miRNAs have opened a new area of investigating the potential therapeutic value in cancer therapy.

Previous studies have examined global messenger RNA (mRNA) and miRNA expression in CRC;^21, 22, 23^ however, only a few have examined the global miRNA and mRNA expression profiling in the same CRC tissue and compared with matched normal tissues.^24^ Thus, we performed global mRNA and miRNA expression profiling in 13 CRC specimens and their matched adjacent normal tissues obtained from Saudi patients. We identified more than 700 potential miRNA-mRNA regulatory networks in CRC controlling various key pathways relevant to CRC development, progression, and therapy failure.

## Results

### Gene expression profiling in CRC

**Figure 1:**
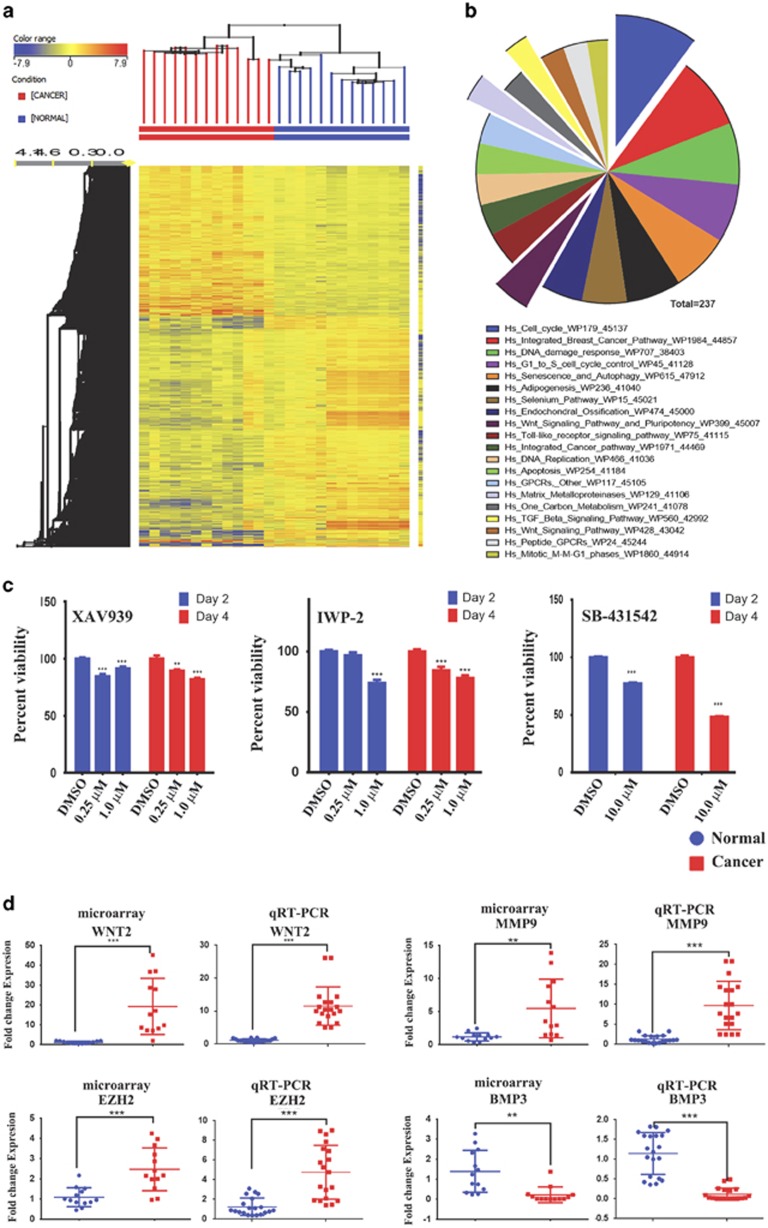
Differentially expressed genes in CRC. (**a**) Hierarchical clustering of 13 CRC and 13 adjacent normal tissue samples based on differentially expressed mRNA levels. Each column represents a sample and each row represents a transcript. Expression level of each gene in a single sample is depicted according to the color scale. (**b**) Pie chart illustrating the distribution of the top 20 pathway designations for the upregulated genes in colon cancer cells. The pie size corresponds to the number of matched entities. (**c**) Inhibition of Wnt pathways using XAV 939 and IWP-2 or TGF-*β* pathway using SB-431542 small-molecule inhibitors led to significant reduction in cell viability in HT115 colon cancer cells. Data are presented as mean±S.E., *n*=24. (**d**) Expression levels of selected genes (*WNT2*, *MMP9*, *EZH2*, and *BMP3*) based on the microarray data and validation of those genes using qRT-PCR (duplicate). **P<0.005; **P<0.0005

Global gene expression profiling was conducted on 13 colon cancer specimens and 13 adjacent normal tissues. The clinical characteristics of patients involved in current study are provided in Table 1. As shown in Figure 1a, hierarchical clustering based on differentially expressed mRNAs revealed clear separation of the two groups where the cancer tissues clustered separately from the normal tissues, except for one branch of the cancer group representing one cancer sample, which was misclassified to the normal group. Using significance analysis, 1273 up- and 1902 downregulated genes were identified (2.0 FC, *P*<0.02; Supplementary Table 1). Pathway analysis on the upregulated genes using GeneSpring GX revealed significant enrichment in several pathways related to cell cycle, DNA damage, matrix metalloproteases, Wnt, and TGF-*β* signaling (Figure 1b and Supplementary Table 2). The Wnt and TGF-*β* signaling pathways are illustrated in Supplementary Figures 1 and 2. To confirm their relevance to CRC, we used small-molecule inhibitors to inhibit the Wnt (XAV939 and IWP-2) or TGF-*β* (SB-431542) signaling in HT115 cells, which led to significant reduction in cell viability (Figure 1c). Selected number of the upregulated (wingless-type MMTV integration site family member 2 (*WNT2*), matrix metallopeptidase 9 (*MMP9*), enhancer of zeste homolog 2 (*EZH2*)) and downregulated (bone morphogenetic protein 3 (*BMP3*)) genes from the microarray data were subsequently validated using quantitative reverse transcription-PCR (qRT-PCR) (Figure 1d). Small-interfering RNA (siRNA)-mediated knockdown of FOXM1 (forkhead box protein M1) and FOXQ1 (forkhead box protein Q1) reduced HT115 cell growth *in vitro* (Supplementary Figure 3), corroborating a biological relevance of the identified genes from the microarray in CRC biology.

### miRNA expression profiling in CRC

**Figure 2:**
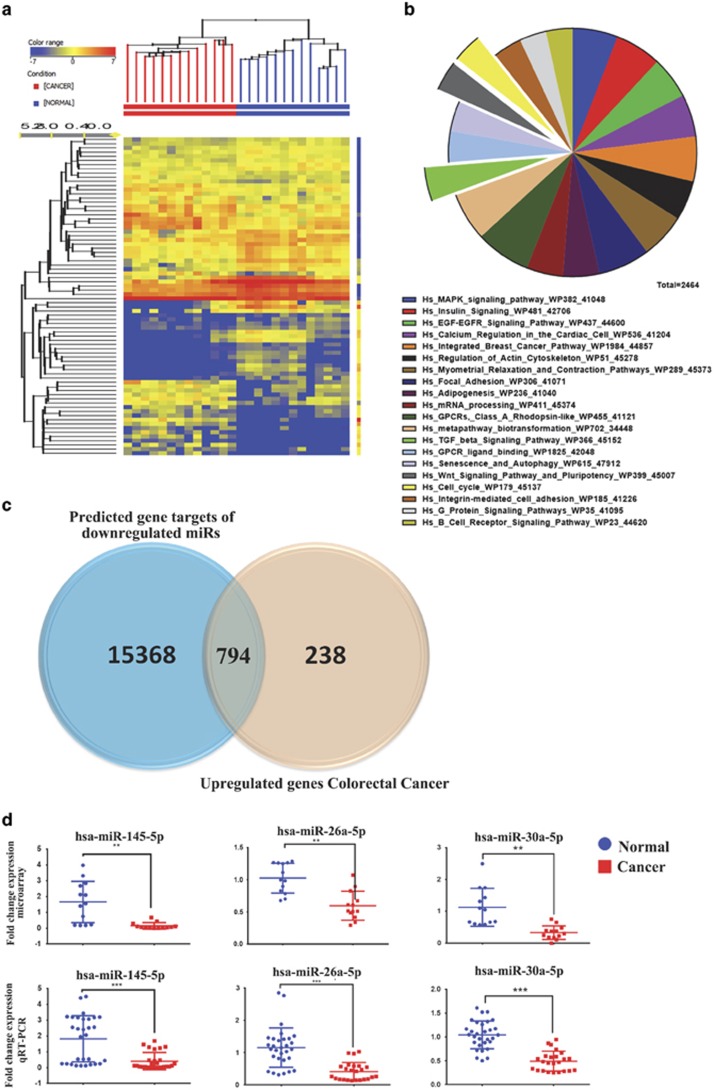
miRNA expression profiling in CRC. (**a**) Hierarchical clustering of 13 colon cancer and 13 normal tissue samples based on miRNA expression levels. Each column represents a sample and each row represents a transcript. Expression level of each miRNA in a single sample is depicted according to the color scale. (**b**) Pie chart illustrating the distribution of the top 20 pathway designations for predicted targets (TargetScan) for the downregulated miRNAs in colon cancer. The pie size corresponds to the number of matched entities. (**c**) Venn diagram depicting the overlap between the predicted gene targets for the downregulated miRNAs (based on TargetScan) *versus* the differentially upregulated genes in CRC identified in the current study. (**d**) Expression levels of selected miRNAs (hsa-miR-145-5p, hsa-miR-26a-5p, and hsa-miR-30-5p) based on microarray data and validation of those miRNAs using Taqman qRT-PCR (duplicate). **P<0.005; ***P<0.0005

To identify potential miRNA-mRNA regulatory networks in CRC, we performed global miRNA expression profiling on the same 13 cancer and adjacent normal samples that were used for mRNA profiling shown in Figure 1. Using significant analysis, we identified 61 significantly downregulated and 42 significantly upregulated miRNAs (1.5-fold change, *P*<0.02; Table 2). Hierarchical clustering of the differentially expressed miRNAs in 13 colon cancer specimes and 13 normal tissues is shown in Figure 2a. The data revealed clear separation of the two groups. We subsequently focused on the downregulated miRNAs and their correlation with the upregulated target genes. Using TargetScan prediction feature in GeneSpring GX software (Agilent Technologies, Santa Carla, CA, USA), 16 157 genes were predicted to be targeted by the identified downregulated miRNAs (Supplementary Table 3). Pathway analysis using the predicted gene targets revealed significant enrichment for several pathways such as cancer, TGF-*β*, FAK (focal adhesion kinase), Wnt, and MAPK (mitogen-activated protein kinase). Top 20 enriched pathways based on TargetScan prediction are shown in Figure 2b. We subsequently focused on the upregulated genes in CRC specimens, which could potentially be regulated by miRNAs found to be downregulated in the same specimens. Crossing the list of predicted gene targets for downregulated miRNAs with the list of the upregulated genes in CRC revealed 794 upregulated genes, which were predicted to be targeted by downregulated miRNAs in CRC (Figure 2c and Supplementary Table 4). The expression levels of selected number of the identified miRNAs from the microarray data (hsa-miR-145-5p, hsa-miR-26a-5p, and hsa-miR-30a-5p) were subsequently validated using Taqman qRT-PCR (Figure 2d).

### Depletion of EZH2/PRC2 complex reduces colon cancer cell proliferation and cell migration

**Figure 3:**
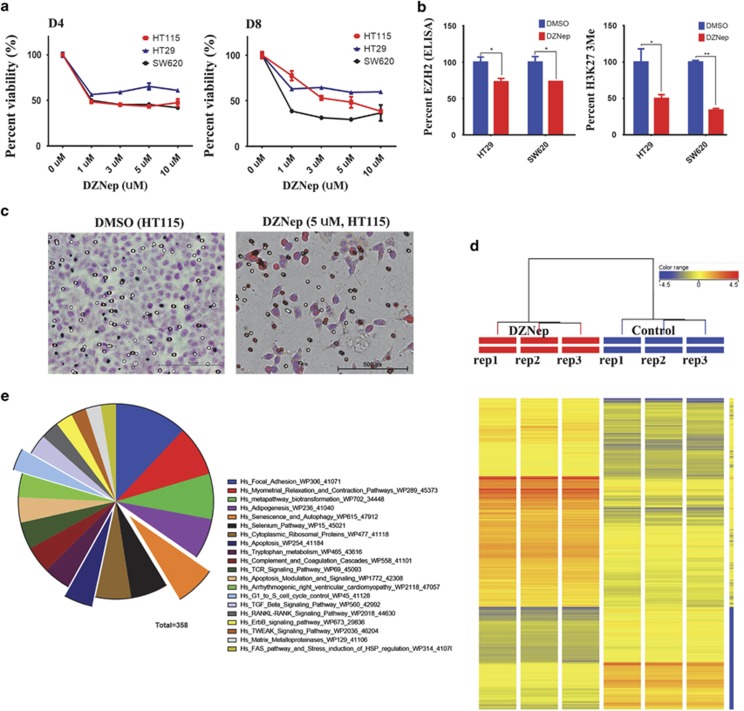
Inhibition of EZH2 using DZNep mediates significant reduction in cell viability and *in vitro* migration in colon cancer cells. (**a**) HT115, HT-29, and SW620 cells were treated with the indicated dose of DZNep, and cell viability was measured on days 4 and 8 posttreatment using the alamarBlue assay. Data are presented as mean±S.E., *n*=8. (**b**) DZNep treatment (5 days) led to significant reduction in EZH2 protein expression in HT-29 and SW620 cells. Similarly, DZNep treatment led to substantial reduction in H3K273me in the colon cancer cells. *P<0.05, *n*=2. (**c**) DZNep treatment led to remarkable reduction in HT115 cell *in vitro* transwell migration. (**d**) Hierarchical clustering of HT115 treated with DZNep (5 *μ*M) compared with controls based on mRNA expression levels. Each column represents one replica. Expression level of each gene in a single replica is depicted according to the color scale. (**e**) Pie chart illustrating the distribution of the top 20 pathway designations for the differentially expressed genes in HT115-DZNep *versus* control. The pie size corresponds to the number of matched entities

Among the identified upregulated genes in our study is EZH2. Elevated expression of EZH2 has been observed in different human cancers,^13, 25, 26, 27^ which we found to be upregulated in CRC in the current study as well (Figure 1d and Supplementary Table 1). To assess the biological ramifications of EZH2 depletion on CRC cancer cells, we treated HT115, HT-29, and SW620 colon cancer cells with 3-deazaneplanocin A (DZNep), a small-molecule inhibitor known to target EZH2 protein, and assessed cell viability on days 4 and 8 posttreatment. As shown in Figure 3a, a significant dose- and time-dependent decrease in colon cancer cell viability was observed, which was associated with a reduction in EZH2 protein expression (Figure 3b, left) and a marked reduction in tri-methylated lysine 27 (H3K27-3me) (Figure 3b, right). Concordant with these data, HT115 cells treated with DZNep also exhibited marked reduction in cell migration as measured using transwell migration assay (Figure 3c). To identify the molecular pathways regulated by EZH2 in CRC, HT115 cells were treated with DZNep to induce reduction in EZH2 and subsequently examined the effects of reduced EZH2 on global gene expression using microarray analysis (Figure 3d). Pathway analysis on the differentially expressed genes revealed multiple enriched pathways including senescence and autophagy, apoptosis, and FAK (Figure 3e and Supplementary Table 5). The senescence and autophagy pathway is illustrated in Supplementary Figure 4, with all matched entities indicated.

### EZH2 is regulated by several miRNAs in CRC

**Figure 4:**
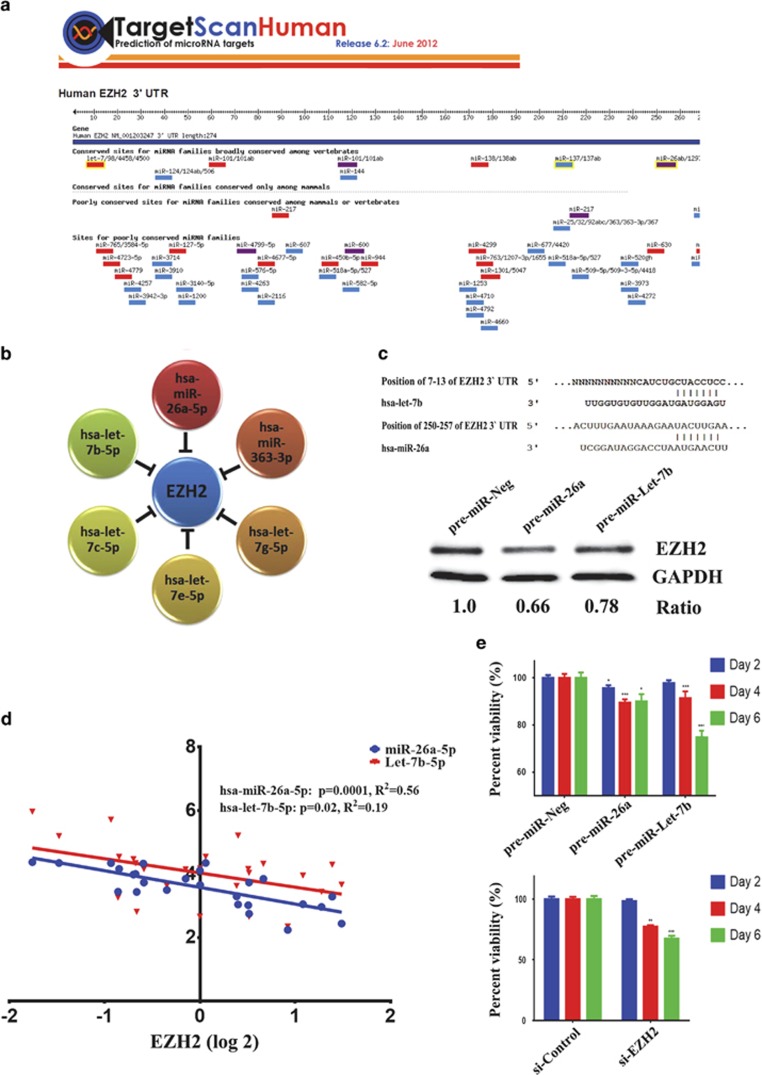
Regulation of EZH2 by hsa-miR-26a and hsa-let-7 family in CRC. (**a**) Schematic presentation showing miRNAs predicted to target EZH2 using TargetScan database. (**b**) EZH2 is predicted to be regulated by several members of the let-7 family, miR-26a-5p, and miR-363-3p, which were downregulated in colon cancer. (**c**) Schematic presentation depicting alignment of Let-7b-5p and miR-26a-5p mature sequence, and the putative binding sites within the 3′-UTR region of the EZH2 mRNA using TargetScan database. The exact positions of the interaction between EZH2 3′-UTR and both miRNA seed regions are indicated. Immunoblotting for EZH2 protein in HT115 transfected with the indicated pre-miRs at 48 h posttransfection. GAPDH was used as a loading control. (**d**) Inverse relationship between EZH2 and hsa-miR-26a-5p and hsa-let-7b-5p expression in 13 CRC and matched normal tissues. (**e**) siRNA-mediated knockdown of EZH2 (lower panel) or exogenous expression of hsa-let7-b-5p and hsa-miR-26a-5p (upper panel) led to significant reduction in cell viability in HT115 colon cancer cell. Data are presented as mean±S.E., *n*=12. *P<0.05; **P<0.005; ***P<0.0005

Our results suggest that EZH2 is involved in multiple aspects of CRC cell biology; therefore, we hypothesized that the elevated expression of EZH2 in CRC could be attributed to the downregulation of miRNAs that targets EZH2. Figure 4a illustrates the map for EZH2 3′-untranslated region (UTR) and the list of miRNAs predicted to target EZH2 based on TargetScan prediction. Among the predicted miRNAs, EZH2 was found to be regulated by six microRNAs (hsa-miR-26a-5p, hsa-Let-7b-5p, hsa-Let-7c-5p, hsa-Let-7e-5p, hsa-Let-7g-5p, and hsa-miR-363-3p), which were downregulated in CRC (Figure 4b and Table 2). We subsequently focused on hsa-miR-26a-5p and hsa-let-7b-5p, as we previously reported those two miRNAs to target EZH2 in nasopharyngeal carcinoma.^13^ The alignment between EZH2 3′-UTR and these two miRNAs is shown in Figure 4c (upper panel). Overexpression of hsa-miR-26a-5p or hsa-let-7b-5p in HT115 cells led to significant reduction in EZH2 protein levels (Figure 4c, lower panel). Interestingly, significant inverse relationship between EZH2 and hsa-miR-26a-5p (*R*^2^= 0.56, *P*=0.0001) and hsa-let-7b-5p (*R*^2^=0.19, *P*=0.02) expression by microarray was observed in the 13 CRC and their matched adjacent normal tissue specimens (Figure 4d), corroborating EZH2 being relevant biological target for these two miRNAs in CRC. Exogenous expression of hsa-miR-26a-5p and hsa-let-7b-5p (Figure 4e, upper panel) led to significant reduction in cell viability, similar to those seen with EZH2 knockdown (Figure 4e, lower panel).

## Discussion

Although a number of previous studies have examined mRNA and/or miRNA expression in CRC,^21, 22, 23, 28, 29^ only few studies have examined global mRNA and miRNA expression in the same clinical samples,^24, 30^ none so far has been conducted in this geographical region. Therefore, the strength of our approach is that it enables the identification of deregulated mRNA-miRNA networks in the same biological specimens.

Our data revealed more than 700 potential miRNA-mRNA regulatory networks in CRC and thus provide circumstantial evidence for the involvement of miRNAs in the pathogenesis of colorectal cancer. In addition, our data provide a comprehensive molecular profiling of CRC in Saudi Arabia and the Middle East.

Several of the deregulated mRNAs and miRNAs identified in the current study have been reported previously, suggesting a common underlying molecular mechanisms leading to CRC pathogenesis regardless of ethnicity. For instance, hsa-miR-135b, hsa-miR-223, hsa-miR-18a, hsa-miR-17, hsa-miR-31, and hsa-miR-21 were upregulated in our study, and were also reported by a previous study.^18^ Similarly, we found hsa-miR-375, hsa-miR-195, hsa-miR-378, hsa-miR-143, hsa-miR-145, hsa-miR-29c, hsa-miR-1, hsa-miR-30c, hsa-miR-30e, hsa-miR-26a, hsa-miR-100, and hsa-miR-338-3p to be downregulated in CRC in our data, which were also reported to be downregulated in colon cancer patients from Northern Europe.^18^

Our data revealed several additional novel miRNAs, which have not been reported previously (Table 2), possibly because of a more comprehensive coverage of miRNAs in the miRNA microarray chips that cover 1205 human miRNAs and used in our study. Our gene expression data revealed multiple deregulated pathways in colon cancer such as cell cycle, DNA damage response, Wnt signaling, and matrix metalloproteases signaling, which is concordant with previous studies implicating these pathways in CRC.^31, 32^ We found that pharmacological inhibition of Wnt or TGF-*β* signaling impaired colon cancer cell proliferation *in vitro* (Figure 1c), which suggests a biological relevance for these pathways in CRC. Several of the identified pathways in CRC were found to be among the predicted targets for miRNAs identified in our study, which suggest a plausible role for the identified miRNAs in the pathogenesis of CRC. We have chosen EZH2, which is a member of the polycomb gene (PcG) family, as it has been implicated in the pathogenesis of a number of other cancer types.^13, 25, 26, 27^ EZH2 is the catalytic subunit of the polycomb repressive complex 2 (PRC2), which is responsible for methylation of lysine 27 on histone H3. This epigenetic modification of H3 is necessary for gene repression through the PRC2 complex. Our current study suggests that EZH2 has a role in the pathogenesis of CRC. Pharmacological inhibition of EZH2 led to significant decrease in H3K27-3me, significant decrease in cell viability, and migration in CRC cells. In addition, siRNA-mediated knockdown of EZH2 exhibited profound effects on colorectal cancer cell growth *in vitro*.

*In silico* prediction has identified several potential miRNAs targeting EZH2 in colon cancer cells, and forced expression of hsa-miR-26a-5p and hsa-let7b-5p phenocopied the effects of EZH2 depletion in CRC cells, supporting a role of the two miRNAs in regulating EZH2 expression in colorectal cancer. Our data are concordant with our previous publication implication hsa-miR-26a-5p and hsa-let-7 family in regulating EZH2 in nasopharyngeal carcinoma.^13^ Interestingly, we observed significant inverse relationship between EZH2 and hsa-miR-26a-5p and hsa-let-7b-5p expression in CRC (Figure 4d), corroborating the biological relevance of this regulatory network in this disease.

In our current study, we have validated one regulatory network for its relevance in CRC cell biology. However, we provided information regarding several other potential regulatory networks (Supplementary Tables 3 and 4) in CRC that remain to be investigated.

## Materials and Methods

### Ethics statement

The clinical study and collection of tissue samples were approved by Institutional Research Ethics Board at the King Saud University College of Medicine (Riyadh, Riyadh, Saudi Arabia).

### Patient and tissue collection

Tissue specimens from 13 fresh-frozen consecutive sporadic CRCs matched with their adjacent normal mucosa were obtained from previously untreated patients who underwent surgical resection at the King Khaled University Hospital (Riyadh, Saudi Arabia). Tumor and their paired normal mucosa were selected by an experienced pathologist and specimens were snap frozen in liquid nitrogen and stored at −80 °C until use. Clinical information of the patients is provided in Table 1.

### Tissue preparation and RNA isolation

Tissues were ground to powder using a mortar and pestle in the presence of liquid nitrogen. RNA was isolated from ~100 to 300 mg of tissue per sample using the Total Tissue RNA Purification Kit from Norgen-Biotek Corp. (Thorold, ON, Canada). The resulting RNA was quantified using NanoDrop 2000 (Thermo Scientific, Wilmington, DE, USA) and the RNA quality and integrity was confirmed using gel electrophoresis.

### Gene expression profiling

Total RNA was extracted as described above using Total RNA Purification Kit (Norgen-Biotek Corp.) according to the manufacturer's instructions. One hundred and fifty nanograms of total RNA was labeled and then hybridized to the Agilent Human SurePrint G3 Human GE 8 × 60 k v16 microarray chip (Agilent Technologies). All microarray experiments were conducted at the Microarray Core Facility (Stem Cell Unit, King Saud University College of Medicine). Normalization and data analyses were conducted using GeneSpring GX software (Agilent Technologies). Pathway analysis were conducted using the Single Experiment Pathway analysis feature in GeneSpring 12.0 (Agilent Technologies) as described before.^33, 34^ Twofold cutoff with *P*<0.02 was used.

### miRNA expression profiling

miRNA expression profiling was conducted on the same 13 RNA samples used for gene expression profiling. Two hundred nanograms of the extracted total RNA was used for RNA labeling and hybridization on to the Agilent Human SurePrint G3 8 × 60k v16 miRNA microarray chip according to the manufacturer's protocol. Data were subsequently normalized and analyzed using GeneSpring GX software (Agilent Technologies). A fold-change of 1.5 with *P*<0.02 was used as cutoff to determine the differentially expressed miRNA in cancer *versus* normal tissues. Target prediction was conducted using a built-in feature in GeneSpring GX based on TargetScan database.

### mRNA and miRNA validation by qRT-PCR

Both mRNA and miRNA expression levels were validated in CRC and normal tissues using qRT-PCR method using the Applied Biosystem (ABI) Detection system. For mRNA expression detection, 2 *μ*g of total RNA was reverse transcribed using High Capacity cDNA Reverse Transcript Kit (Part No: 4368814; ABI) according to the manufacturer's protocol. Relative levels of mRNA were determined from cDNA using real-time PCR (Applied Biosystems StepOnePlus Real-Time PCR Systems). Primer sequences used in the current study were: WNT2 (F), 5′-GCGCATTTGTGGATGCAAAG-3′ and (R), 5′-ACCGCTTTACAGCCTTCCTG-3′ BMP3 (F), 5′-GCAGCAGCAGAAACTCTTGAAA-3′ and (R), 5′-AGACACTGGACAACTCAGGC-3; MMP9 (F), 5′-CGGTTTGGAAACGCAGATGG-3′ and (R), 5′-TGGGTGTAGAGTCTCTCGCT-3′ and EZH2 (F), 5′-GCGCGGGACGAAGAATAATCAT-3′ and (R), 5′-TACACGCTTCCGCCAACAAACT-3′. For miRNA expression detection, 10 ng of total RNA was reverse transcribed using TaqMan MicroRNA Reverse Transcription Kit (Part No: 4366596; ABI), and relative miRNA expression levels were determined using TaqMan Universal Master Mix II, No UNG (Part No: 4440040), and hsa-miR-26a-5p (Assay ID: 000405), hsa-miR-30a-5p (Assay ID: 000417), and hsa-miR-145-5p (Assay ID: 002278) from ABI. The relative expression level was calculated using –ΔΔCT. RNU44/RNU48 was used as an endogenous control for miRNA expression, whereas *β*-actin was used as an endogenous control for mRNA expression.

### Cell culture

Human colorectal cell lines HT-29, HT115, and SW620 were purchased from CLS Cell Lines Service GmbH (Eppelheim, Germany). The cell line was incubated in Dulbecco's modified Eagle's medium supplemented with d-glucose 4500 mg/l, 4 mM l-glutamine and 110 mg/l sodium pyruvate, 10% fetal bovine serum, 1x penicillin–streptomycin (Pen-Strep) and non-essential amino acids (all purchased from Gibco-Invitrogen, Waltham, MA, USA). Colon cancer cell line HT-29, HT115, and SW620 were treated with 5 *μ*M DZNep small-molecule inhibitor of EZH2 (Sigma, St. Louis, MO, USA). Pharmacological inhibition of Wnt pathway was conducted using XAV939 (0.25 and 1 *μ*M) and IWP-2 (1 and 5 *μ*M). Inhibition of TGF-*β* pathway was conducted as we previously described using 10 *μ*M SB-431542 (Sigma, St. Louis, MO, USA).^34^ Assays were carried out with appropriate controls such as dimethyl sulfoxide. Briefly, 10 000 cells were cultured in a 96-well plate and cell viability was measured at the indicated time points using the alamarBlue (BUF012B; AbD Serotec, Kidlington, UK) assay.

### Transfection

The pre-miR-negative control, hsa-miR-26a-5p, hsa-let-7b-5p, siControl, and siEZH2 were purchased from Applied Biosystems (Invitrogen, Carlsbad, CA, USA). Transfection was performed using reverse transfection approach as described before.^14^ Briefly, 30 nM (final) pre-miRs or 30 nM (final) siRNA was diluted in 50 *μ*l of Opti-MEM (11058-021; Gibco, Carlsbad, CA, USA), whereas 1 *μ*l of Lipofectamine 2000 (Part No: 52758; Invitrogen) were diluted in 50 *μ*l OPTI-MEM. The diluted pre-miR, siRNA, and Lipofectamine 2000 were mixed and incubated at ambient temperature for 20 min. Twenty microliters of transfection mixture was added to the plate and subsequently 10 000 cells in transfection medium (routine culture medium without antibiotics) were added to each well in 60 *μ*l volume. Every experiment was performed in 10 replicates in 96-well cell culture plates with the appropriate controls. The experiment was repeated at least two times. Plates were incubated for the indicated time points, and proliferation or growth inhibition was assessed using the alamarBlue (BUF012B; AbD Serotec) assay.

### Histone methylation quantification (global H3K27 methylation assay)

Colon cancer cell lines HT-29 and SW620 were treated with 5 *μ*M DZNep small-molecule inhibitor (cat. no. 13828; Cayman Chemical, Ann Arbor, MI, USA) for 5 days. Histones were extracted and quantified (Colorimetric) from control and drug-treated cells using the EpiQuik Global Histone H3-K27 Assay Kit (*P*-3020; Epigentek, Farmingdale, NY, USA) according to the manufacturer's Instructions. Data were normalized on total histone H3 quantified using the EpiQuik Total Histone H3 (Methylated H3-K27 Control) Quantification Kit (Colorimetric, Epigentek).

### EZH2 quantification by ELISA

Colon cancer cell lines HT-29 and SW620 were treated with 5 *μ*M DZNep drug (13828; Cayman Chemical, Ann Arbor, MI, USA) for 5 days. *In vitro* quantitative measurement of EZH2 was executed by sandwich enzyme immune assay (EZH2, ELISA (enzyme-linked immunosorbent assay) Kit; MyBioSource Inc., San Diego, CA, USA). Both controls and drug-treated cells were lysed and analyzed with respective standards according to the manufacturer's instructions.

### Immunoblotting

HT115 cells were transfected with the indicated pre-miR. Forty-eight hours later, cells were lysed using RIPA buffer (Norgen-Biotek Corp.) containing 1 × Halt Protease Inhibitor Cocktail (Pierce Inc., Rockford, IL, USA). Thirty micrograms of total protein were run and blotted using the Bio-Rad V3 Western work flow system according to the manufacturer's recommendation. Immunoblotting was conducted using anti-EZH2 rabbit polyclonal antibody (D2C9, 1 : 1000 dilution; Cell Signaling, Beverly, MA, USA) overnight at 4 °C. Horseradish peroxidase (HRP)-conjugated goat anti-rabbit (cat. no. 7074, 1 : 3000 dilution; Cell Signaling) was used as the secondary antibody, whereas HRP-conjugated anti-GAPDH (glyceraldehyde-3-phosphate dehydrogenase) antibody (ab9482, 1 : 10000; Abcam, Cambridge, MA, USA) was used as the loading control.

### Statistical analysis

Statistical analyses and graphing were performed using Microsoft excel 2010 and GraphPad Prism 6.0 software (GraphPad, San Diego, CA, USA). *P*-values were calculated using the two-tailed *t*-test. Pearson's correlation was used to assess the correlation between EZH2, hsa-miR-26a, and hsa-let-7b-5p expression using the GraphPad Prism software.

## Figures and Tables

**Table 1:** Clinical characteristics for the patients used in the current study

**No.**	**Age (years)**	**Sex**	**Site of cancer**	**Adenocarcinoma**	**Histological grade**	**Clinical stage**	**TNM classification**		
1	70	Male	Sigmoid	Yes	G2	Stage III	T4	N1	M1
2	47	Male	Sigmoid	Yes	G2	Stage III	T3	N1	M1
3	50	Male	Colon	Yes	G2	Stage II	T3	N0	M0
4	56	Female	Rectum	Yes	G2	Stage II	T3	N0	M0
5	59	Male	Colon and rectum	Yes	G2	Stage III	T3	N2	M1
6	72	Male	Sigmoid	Yes	G2	Stage II	T3	N0	M0
7	70	Male	Rectum	Yes	G2	Stage III	T3	N2	M1
8	68	Female	Colon	Yes	G2	Stage II	T4	N0	M0
9	57	Female	Rectosigmoid	Yes	G2	Stage III	T3	N2	M1
10	61	Female	Sigmoid	Yes	G2	Stage II	T3	N0	M0
11	30	Female	Sigmoid	Yes	G2	Stage III	T2	N1	M1
12	57	Male	Sigmoid	Yes	G3	Stage III	T3	N1	M1
13	59	Female	Sigmoid	Yes	G2	Stage III	T3	N1	M1

Abbreviations: M, presence of distant metastasis; N, degree of spread to regional lymph nodes; T, size or direct extent of the primary tumor

Classification of malignant tumors (TNM)

**Table 2:** Differentially expressed miRNAs between colorectal cancer specimens and normal tissues

**Updated systematic name**	**FC C versus N**	**Log FC C versus N**	**Regulation C versus N**	**miRBase accession no.**
*Downregulated miRs*				
hsa-miR-133b	−226.08115	−7.820697	Down	MIMAT0000770
hsa-miR-378a-5p	−110.78563	−6.791627	Down	MIMAT0000731
hsa-miR-139-5p	−84.79849	−6.4059668	Down	MIMAT0000250
hsa-miR-133a	−84.60821	−6.4027257	Down	MIMAT0000427
hsa-miR-145-3p	−74.17002	−6.2127643	Down	MIMAT0004601
hsa-miR-1	−58.168026	−5.8621545	Down	MIMAT0000416
hsa-miR-30e-3p	−32.476025	−5.021303	Down	MIMAT0000693
hsa-miR-143-5p	−31.099096	−4.958801	Down	MIMAT0004599
hsa-miR-29c-5p	−28.070925	−4.8110046	Down	MIMAT0004673
hsa-miR-486-5p	−21.722443	−4.4411144	Down	MIMAT0002177
hsa-miR-99a-5p	−20.735474	−4.374029	Down	MIMAT0000097
hsa-miR-139-3p	−20.527025	−4.3594527	Down	MIMAT0004552
hsa-miR-363-3p	−17.026367	−4.089699	Down	MIMAT0000707
hsa-miR-490-5p	−16.500505	−4.0444384	Down	MIMAT0004764
hsa-miR-129-1-3p	−16.097223	−4.00874	Down	MIMAT0004548
hsa-miR-4328	−15.330928	−3.938373	Down	MIMAT0016926
hsa-miR-3675-3p	−14.637202	−3.871568	Down	MIMAT0018099
hsa-miR-145-5p	−13.633393	−3.7690728	Down	MIMAT0000437
hsa-miR-28-3p	−10.397909	−3.3782215	Down	MIMAT0004502
hsa-miR-1267	−10.369238	−3.374238	Down	MIMAT0005921
hsa-miR-3679-3p	−9.957987	−3.315854	Down	MIMAT0018105
hsa-miR-1227	−9.898095	−3.3071508	Down	MIMAT0005580
hsa-miR-99b-5p	−8.877857	−3.1502116	Down	MIMAT0000689
hsa-miR-4324	−8.527493	−3.0921216	Down	MIMAT0016876
hsa-miR-100-5p	−8.12212	−3.0218563	Down	MIMAT0000098
hsa-miR-143-3p	−7.537136	−2.9140165	Down	MIMAT0000435
hsa-miR-634	−7.271931	−2.8623385	Down	MIMAT0003304
hsa-miR-129-2-3p	−6.9524083	−2.7975128	Down	MIMAT0004605
hsa-miR-490-3p	−6.2970576	−2.6546779	Down	MIMAT0002806
hsa-miR-497-5p	−5.41665	−2.4374008	Down	MIMAT0002820
hsa-miR-28-5p	−5.246121	−2.391251	Down	MIMAT0000085
hsa-miR-378a-3p	−5.230102	−2.3868392	Down	MIMAT0000732
hsa-miR-30a-5p	−5.11498	−2.3547287	Down	MIMAT0000087
hsa-miR-125b-5p	−3.6129825	−1.8531903	Down	MIMAT0000423
hsa-miR-195-5p	−3.1166792	−1.6400096	Down	MIMAT0000461
hsa-miR-375	−3.0833967	−1.6245205	Down	MIMAT0000728
hsa-miR-125a-5p	−2.7951596	−1.4829307	Down	MIMAT0000443
hsa-miR-23b-3p	−2.5185568	−1.3325973	Down	MIMAT0000418
hsa-miR-30c-5p	−2.4603393	−1.2988573	Down	MIMAT0000244
hsa-miR-548c-5p	−2.393883	−1.2593527	Down	MIMAT0004806
hsa-miR-150-5p	−2.1257915	−1.0880002	Down	MIMAT0000451
hsa-miR-140-3p	−1.9857309	−0.98967016	Down	MIMAT0004597
hsa-miR-27b-3p	−1.8863192	−0.91557384	Down	MIMAT0000419
hsa-miR-3653	−1.8339884	−0.8749845	Down	MIMAT0018073
hsa-miR-320a	−1.8225296	−0.86594224	Down	MIMAT0000510
hsa-let-7c	−1.821517	−0.86514044	Down	MIMAT0000064
hsa-miR-26a-5p	−1.7855949	−0.8364048	Down	MIMAT0000082
hsa-let-7b-5p	−1.7462178	−0.80423355	Down	MIMAT0000063
hsa-miR-365a-3p	−1.7350858	−0.79500705	Down	MIMAT0000710
hsa-miR-320e	−1.724407	−0.78610027	Down	MIMAT0015072
hsa-miR-320d	−1.7153007	−0.77846146	Down	MIMAT0006764
hsa-miR-320b	−1.7146243	−0.7778925	Down	MIMAT0005792
hsa-miR-361-5p	−1.687753	−0.7551037	Down	MIMAT0000703
hsa-miR-30e-5p	−1.6607047	−0.73179555	Down	MIMAT0000692
hsa-miR-4313	−1.6525247	−0.72467184	Down	MIMAT0016865
hsa-miR-320c	−1.6258739	−0.7012154	Down	MIMAT0005793
hsa-miR-29c-3p	−1.6134787	−0.69017446	Down	MIMAT0000681
hsa-let-7b-3p	−1.6106501	−0.68764305	Down	MIMAT0004482
hsa-miR-149-5p	−1.5800085	−0.6599323	Down	MIMAT0000450
hsa-let-7 g-5p	−1.5541947	−0.6361673	Down	MIMAT0000414
hsa-miR-423-5p	−1.5068012	−0.5914891	Down	MIMAT0004748
				
*Upregulated miRs*				
hsa-miR-135b-5p	348.6332	8.445566	Up	MIMAT0000758
hsa-miR-96-5p	133.09624	7.056326	Up	MIMAT0000095
hsa-miR-424-5p	85.915306	6.4248433	Up	MIMAT0001341
hsa-miR-18a-5p	55.54994	5.7957134	Up	MIMAT0000072
hsa-miR-31-5p	37.379402	5.2241716	Up	MIMAT0000089
hsa-miR-224-5p	34.20287	5.0960455	Up	MIMAT0000281
hsa-miR-1290	31.966501	4.998489	Up	MIMAT0005880
hsa-miR-130b-3p	27.430082	4.777687	Up	MIMAT0000691
hsa-miR-532-5p	23.844732	4.5755987	Up	MIMAT0002888
hsa-miR-3648	22.774778	4.509365	Up	MIMAT0018068
hsa-miR-203	18.44064	4.204817	Up	MIMAT0000264
hsa-miR-21-3p	17.213509	4.105469	Up	MIMAT0004494
hsa-miR-660-5p	15.831627	3.9847376	Up	MIMAT0003338
hsa-miR-182-5p	15.797628	3.981636	Up	MIMAT0000259
hsa-miR-95	15.297556	3.9352293	Up	MIMAT0000094
hsa-miR-7-5p	12.604156	3.6558275	Up	MIMAT0000252
hsa-miR-3127-5p	12.075171	3.5939717	Up	MIMAT0014990
hsa-miR-18b-5p	11.8246565	3.5637264	Up	MIMAT0001412
hsa-miR-622	10.575571	3.4026637	Up	MIMAT0003291
hsa-miR-3156-5p	10.303987	3.3651307	Up	MIMAT0015030
hsa-miR-652-3p	9.751009	3.2855515	Up	MIMAT0003322
hsa-miR-371a-5p	8.452332	3.0793493	Up	MIMAT0004687
hsa-miR-183-5p	7.5481267	2.9161186	Up	MIMAT0000261
hsa-miR-491-5p	7.534784	2.913566	Up	MIMAT0002807
hsa-miR-3198	6.5412736	2.7095716	Up	MIMAT0015083
hsa-miR-19a-3p	4.7948313	2.26148	Up	MIMAT0000073
hsa-miR-210	4.494275	2.1680884	Up	MIMAT0000267
hsa-miR-1246	4.1132383	2.0402746	Up	MIMAT0005898
hsa-miR-21-5p	3.5413451	1.8242974	Up	MIMAT0000076
hsa-miR-3647-5p	2.8310268	1.5013254	Up	MIMAT0018066
hsa-miR-20a-5p	2.7826865	1.4764783	Up	MIMAT0000075
hsa-miR-17-5p	2.56975	1.361628	Up	MIMAT0000070
hsa-miR-1274a	2.368945	1.2442446	Up	MIMAT0005927
hsa-miR-19b-3p	2.231824	1.1582232	Up	MIMAT0000074
hsa-miR-491-3p	2.1232426	1.0862693	Up	MIMAT0004765
hsa-miR-663a	2.0279565	1.0200267	Up	MIMAT0003326
hsa-miR-20b-5p	1.9541535	0.9665438	Up	MIMAT0001413
hsa-miR-106b-5p	1.9268446	0.94624025	Up	MIMAT0000680
hsa-miR-3651	1.8909732	0.91912895	Up	MIMAT0018071
hsa-miR-642b-3p	1.8161368	0.86087286	Up	MIMAT0018444
hsa-miR-221-3p	1.698573	0.76432323	Up	MIMAT0000278
hsa-miR-425-5p	1.6854963	0.75317353	Up	MIMAT0003393

## Abbreviations

CRC: colorectal cancer
RNA: ribonucleic acid
miR/miRNA: microRNA
mRNA: messenger RNA
siRNA: small interfering RNA
WNT2: wingless-type MMTV integration site family member 2
EZH2: enhancer of zeste homolog 2
let-7: lethal-7
FOXM1: forkhead box protein M1
FOXQ1: forkhead box protein Q1
MMP-9: matrix metallopeptidase 9
BMP2: bone morphogenetic protein 2
BMP3: bone morphogenetic protein 3
qRT-PCR: quantitative reverse transcription polymerase chain reaction
H3k27me3: trimethylated histone H3 on lysine 27
TGF-beta: transforming growth factor-*β*
FAK: focal adhesion kinase
MAPK: mitogen-activated protein kinase
DZNep: 3-deazaneplanocin A
PcG: polycomb group
PRC2: polycomb repressive complex 2
UTR: untranslated region
HRP: horseradish peroxidase
GAPDH: glyceraldehyde 3-phosphate dehydrogenase
ELISA: enzyme-linked immunosorbent assay
ABI: Applied Biosystem
