# Supplementary material for: Genome-wide mRNA and miRNA expression profiling reveal multiple regulatory networks in colorectal cancer
Source: Cell Death Dis. 2015 Jan 22;6(1):e1614–. doi: 10.1038/cddis.2014.556 (PMC4669754; doi:10.1038/cddis.2014.556)
Supplement: Supplementary Figure 2 [file cddis2014556x2.pdf]

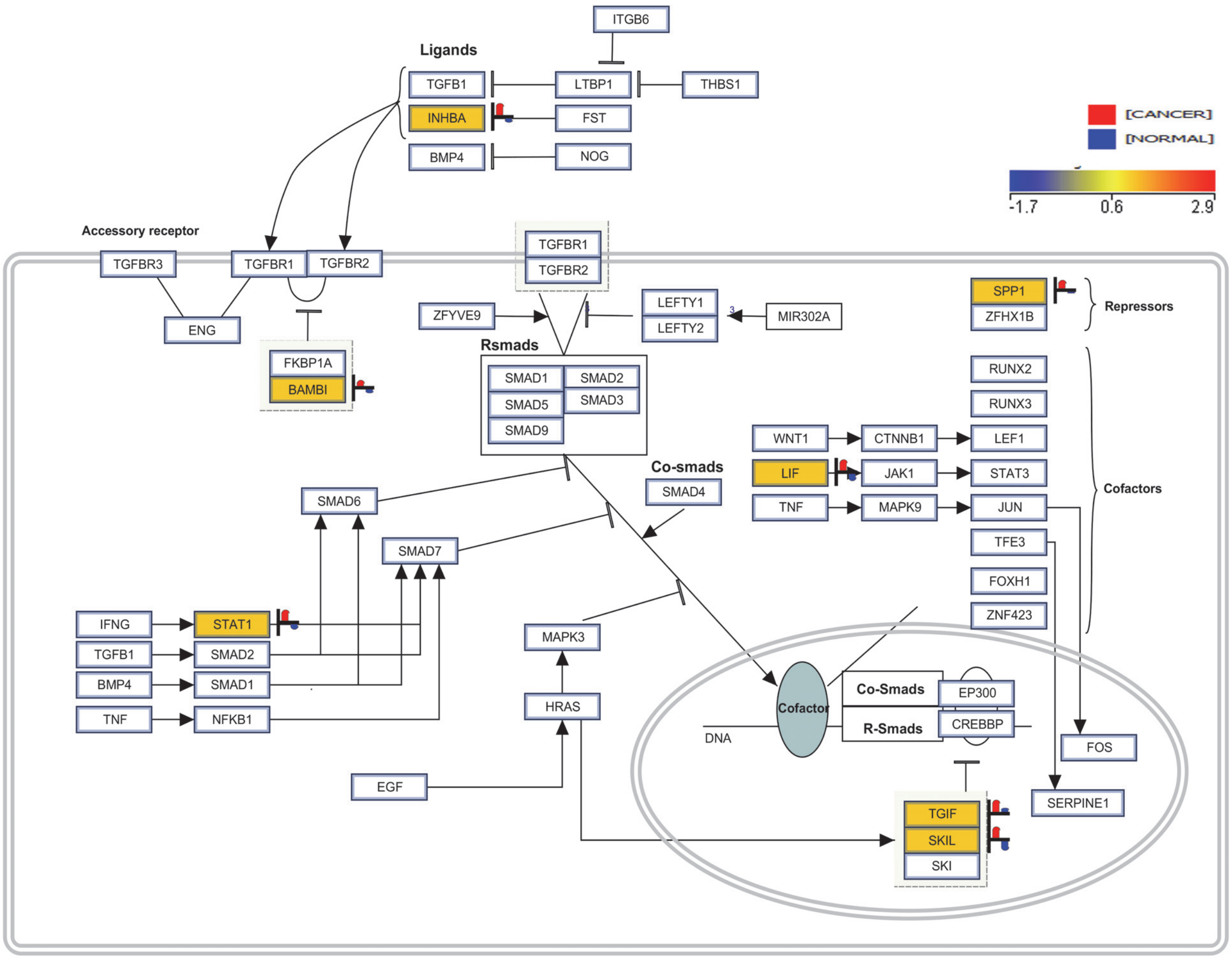


**Supplementary Figure 2.** Illustration of the TGF-beta signaling pathway indicating the matched genes that were upregulated in colorectal cancer.
