# Supplementary material for: Genome-wide mRNA and miRNA expression profiling reveal multiple regulatory networks in colorectal cancer
Source: Cell Death Dis. 2015 Jan 22;6(1):e1614–. doi: 10.1038/cddis.2014.556 (PMC4669754; doi:10.1038/cddis.2014.556)
Supplement: Supplementary Figure 3 [file cddis2014556x3.pdf]

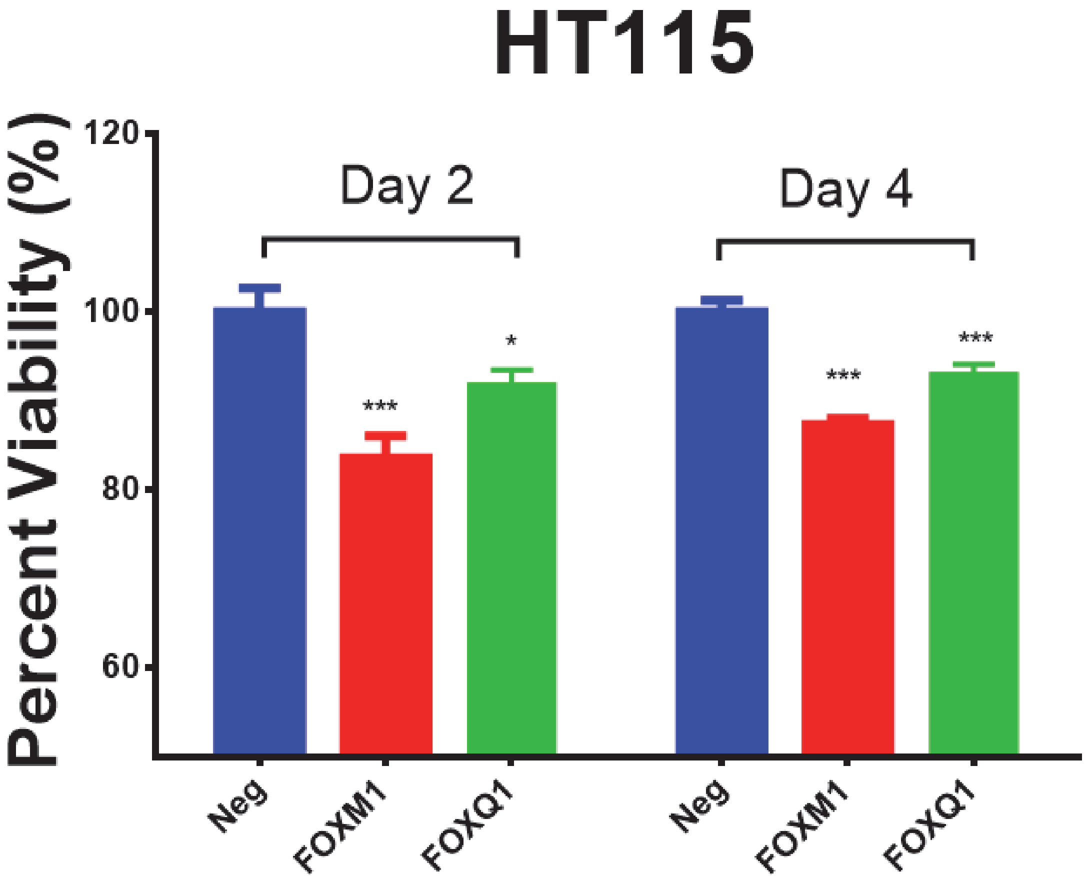

Supplementary Figure 3. siRNA-mediated silencing of FOXM1 and FOXQ1 identified in the microarray data reduced HT115 cell growth *in vitro* on days 2 and 4 post transfection (30 nM) with the indicated siRNAs. Data are presented as mean ± S.E.M, n= 12.
