# Supplementary material for: Genome-wide mRNA and miRNA expression profiling reveal multiple regulatory networks in colorectal cancer
Source: Cell Death Dis. 2015 Jan 22;6(1):e1614–. doi: 10.1038/cddis.2014.556 (PMC4669754; doi:10.1038/cddis.2014.556)
Supplement: Supplementary Figure 4 [file cddis2014556x4.pdf]

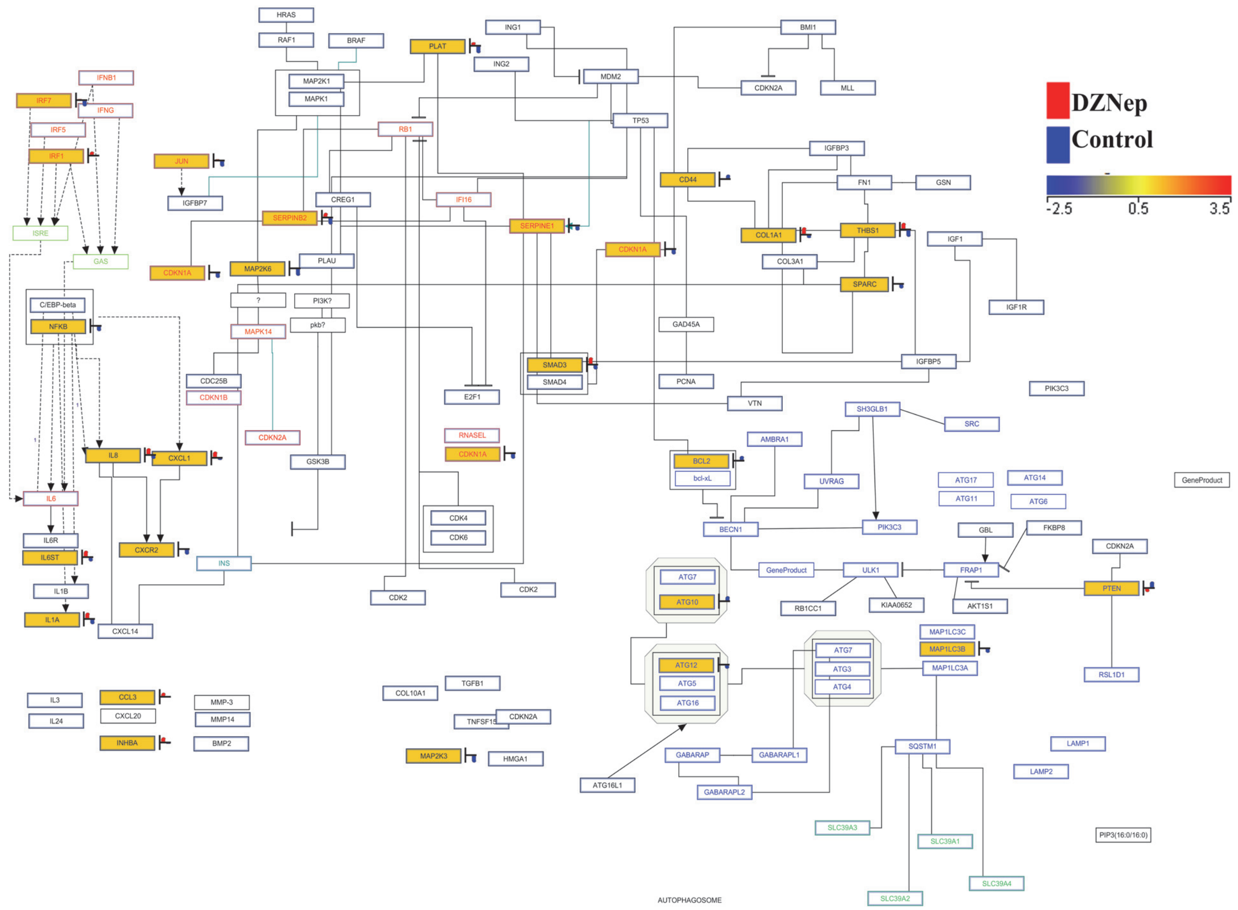

Supplementary Figure 4. Illustration of the senescence and autophagy signaling pathway indicating the matched genes that were differentially expressed in DZNep treated HT115 cells microarray data.
